# Analysis of the Factors Influencing Enterprise and Government Participation in the Medicines Patent Pool Based on System Dynamics Model

**Published:** 2018-10

**Authors:** Jinjing ZENG, Wende ZHANG, Qingming TANG

**Affiliations:** 1. School of Economics and Management, Fuzhou University, Fuzhou, China; 2. Library, Fujian Agriculture and Forestry University, Fuzhou, China; 3. Toyota Technological Institute at Chicago, Chicago, IL, USA

**Keywords:** Medicines patent, Patent pool, Medicine accessibility, System dynamics, Intellectual property

## Abstract

**Background:**

The participation of enterprises and governments in the Medicines Patent Pool (MPP) improves the disease management levels by enhancing the accessibility of medical resources. Non-participation of the stakeholders restricts the development of the MPP. Hence, systematic analysis of the key factors influencing MPP participation is necessary.

**Methods:**

A system dynamics model of the market before and after enterprises and governments join the MPP was constructed by considering the economic benefits of both stakeholders. The effects of generic drug prices, royalty rates, pooling subsidies, and enterprise scale on the relevant benefit difference were analyzed. Data from the China Medical and Economic Network for the period 2003–2016, as well as the 2017 annual report of Celgene Corporation, were used as test data.

**Results:**

The proper pooling subsidy coefficient ranges between 0.05 and 0.08 when the generic drug price ratio and royalty rate are lower than 36% and 34%, respectively. These factors could enhance the willingness of both stakeholders to join the MPP. Initial R&D investments and the relative drug patent intensity of enterprises respectively exert positive and negative impacts on their willingness to join the MPP.

**Conclusion:**

To encourage stakeholders to join the MPP, generic drug prices should be lowered, license fees and subsidies should be adjusted appropriately, and the R&D scale and strength of original drug enterprises should be taken into account. The research results provide a reference for formulating the rules of MPP and other policies aiming to facilitate the sharing and innovation of medical resources.

## Introduction

The general public is highly concerned about increasing treatment options for infectious and chronic diseases, as well as the popularity and accessibility of new essential medicines (1–2). The Medicines Patent Pool (MPP) authorizes qualified generic drug enterprises to produce newly developed medicines through cooperation with pharmaceutical companies and international organizations (3). After nearly 10 years of development, the MPP has played a prominent role in promoting the treatment of AIDS, tuberculosis, and hepatitis and aims to include antineoplastic drugs among its concerns (4). The MPP not only facilitates the improvement and R&D of pharmaceutical formulations and provides more treatment opportunities to patients but also reduces public health expenditures (5). Enterprises joining the MPP provide patented medicines, and this fact is a prerequisite for the construction and operation of the organization. As the primary beneficiary of the MPP, governments, who represent the public, are the most capable agencies to balance the returns and risks to R&D of original drug enterprises after they join the MPP. As governments and enterprises join the organization, the MPP is able to provide more-affordable and high-quality medicines to the public (6), which can help increase public health services and quality of life (7). In the current environment, since a conflict of interest between enterprises and governments, who serve as drug sellers and buyers, respectively, simultaneous participation of these stakeholders in the MPP is unlikely, resulting in a limited member size and drug coverage of the MPP.

Existing studies have mainly explored the factors influencing the decision of enterprises and government to join the MPP from the perspectives of policies, laws, and moral consensus. For example, Dionisio et al. and Balasubramaniyan believed that national policies and MPP rules were the main factors influencing the decision of enterprises to join the MPP (8–9). Some studies revealed that, since stakeholders join the MPP by choice, their participation in the organization was essentially influenced by economic factors, including enterprise profits and government medical expenditures (10–12). The existing research has discussed the influences of sharing medical resources on economic benefits of both enterprise and government (13–14). However, changes in economic benefits before and after these stakeholders join the MPP have not been considered comprehensively, and quantitative analysis of specific drugs and scenarios is lacking.

Considering the current state of research in this field, the present study quantitatively analyzes the economic factors influencing government and enterprise participation in the MPP and provides a new approach to promote research on the MPP joining rate. By balancing the economic interests of stakeholders, this study constructs a system dynamic model of the drug market after both groups join the MPP. This model can be used by MPP organizers to estimate the costs entailed when enterprises and governments join the MPP and formulate admission rules. The research results are applicable to government budgets for medicines and provide the government with a reference for formulating medical policies.

### Literature review

Studying the economic factors that encourage enterprises and governments to join the MPP can help expand the existing MPP scale, which may enhance disease treatment and public health service levels.

The relationships among market share, third-party investments, license fees, and willingness of an enterprise to join the MPP have been widely discussed. By discussing MPP’s construction details, Sukkar pointed out that “new larger market share” was the main factor influencing original drug enterprises’ decision (15). The market share of drugs was mainly influenced by the drug price. Based on the two-way fixed effects model, Skipper et al. reported that public selection of drugs was mainly determined by the price effect of drugs (16). Meanwhile, the generic drug price dominated the price in the market (5). The Organization for Economic Cooperation and Development (OECD) reported that predictions of returns from early R&D costs was the key factor which original drug enterprises concerned (13). To reduce enterprises’ misgivings about significant R&D investment losses, the World Health Organization proposed separating subsidies and R&D costs (17). Scholars discovered that license fees and enterprise size could influence the decisions of stakeholders in traditional MPP. For instance, Hytönen simulated the electronics patent pool and found that overly high license fees caused authorized enterprises to quit (18). Yue analyzed the patent strategies of enterprises based on the economic game theory model and found that non-monopoly innovative enterprises were more likely to join (19). Layne-Farrar et al. agreed with this notion (20). Inspired by these previous findings, the present study selected generic drug price, pooling subsidy, royalty rate, and enterprise scale as key factors influencing an enterprise’s decision to the MPP.

The decision of governments to join the MPP is mainly influenced by public health expenditures. Miguel disclosed that the main goal of governments was to decrease the drugs price, by summarizing the flexibility of public health policies in intellectual property law and pricing strategies (21). Juneja estimated the expenditures saved by governments for anti-retroviral drugs through the MPP and found that generic drug price was the key factor (22). Costa et al. agreed with this finding (23). Hoen suggested separating R&D cost from product price to cope with the challenges preventing stakeholders from joining the MPP and analyzed the necessity of government subsidies (24). Obviously, there is a cost limit on government medical expenditure (25). Therefore, generic drug prices and pooling subsidies may be regarded as key factors influencing governments’ participation in the MPP.

System dynamics is an effective method to analyze causal relationships in dynamic processes and describe the interactions of state variables in the system. This method has been applied in several studies on drug supply chains. For example, Paich et al. analyzed the drug market by using a system dynamics model of the selection behaviors of doctors (26). Bam et al. constructed a system dynamics model to evaluate the validity of amikacin downstream supply chain policies (27). The model has not been applied to analyze the factors influencing the decision of enterprises and governments to join the MPP. Since changes in the benefits of these stakeholders in the market environment were relatively complicated, system dynamics, which can capture the nonlinear relationships of variables to describe stakeholders’ behaviors, can be appropriate.

The literature review revealed that existing studies mainly focused on qualitative analyses of the economic factors influencing enterprises’ or governments’ decision to join the MPP. In this study, a system dynamics model of the drug market before and after stakeholders join the organization is constructed by balancing their economic benefits and the key conditions are analyzed. Two types of enterprises (i.e., original drug enterprises and generic drug enterprises) and governments are involved. Whereas enterprises only decide to join the MPP because of profits, governments participate when their medical expenditures after entry decrease. In this study, only the economic benefits brought by drug price changes are considered. In contrast to previous research, quantitative analysis of the factors influencing enterprises’ and governments’ decision to join was carried out by balancing the economic benefits to both groups.

## Methods

### Data resources and description Data resources

The test data were mainly sourced from the China Medical and Economic network (MENET) database, National Bureau of Statistics of the People’s Republic of China, and the 2017 annual report of Celgene Corporation (USA), which provides information for the period 2003– 2017 on the sales volumes of drugs, market shares, initial R&D investments of enterprises, and disease morbidity statistics. The medicinal purchases of hospitals in 16 cities (i.e., Beijing, Changsha, Chengdu, Guangzhou, Haerbin, Hangzhou, Jinan, Nanjing, Shanghai, Shenyang, Shijiazhuang, Tianjin, Wuhan, Xi’an, Zhenzhou and Chongqing) in China were studied. In this study, data on lenalidomide were used to test the model. Lenalidomide is an anti-multiple myeloma (MM) drug developed by Celgene Corporation. According to MENET, lenalidomide was launched in the USA in June 2005 and in China in 2013. One of the core patents of this drug expired in 2017. In December, 2017, lenalidomide was priced at $174/25 mg after negotiation with health insurance in China; this number was only 40% of the previous price. The monthly dosage was 21 days × 25 mg. Therefore, the annual medical expense for lenalidomide was set to $80,000 before 2017 and $32,000 after 2017.

### Data description

The main variables and parameter values of the constructed model reflect the main factors influencing the decision of enterprises and governments to join the MPP. In this study, three relevant experts were interviewed. The concepts of the subsystem and essential variables in the model were determined, and the model was revised repeatedly. Vensim PLE was applied for model simulation. According to data on MENET (http://www.menet.com.cn/) and investigation statistics, the average period from drug launching and to patent expiration is 12 years, and generic drugs are launched about 2 years after expiration of a drug patent. Therefore, the unit step length of the model was set to 2 months and the total simulation time span was set to 16 years, starting from the launching of the original drug. Vecade, another drug used to treat MM developed by Johnson & Johnson, was launched in China in 2005. According to Hoen, the average initial R&D investments of new drugs in 2002 and 2012 were $0.8 billion and $1.5 billion, respectively (24). Combining these reports with investigation data, the initial R&D investments of original drug enterprises in and out of the MPP in 2015 would be $1 billion. The Chinese population was used as the basis of a healthy population, information for which could be collected from the National Bureau of Statistics. According to estimations by Dr. Zhang Yizhuo of Tianjin Tumor Hospital (28), the morbidity of MM is 2–4/100,000. According to previous investigations, the generic drug price of lenalidomide in China is about 40% of the original drug price. The descriptions and values of other parameters are listed in Table 1.

**Table 1:** List of parameters used in the model

***Parameter***	***Description***	***Value***	***Source***
σ	Proportion of affordable population	32%	National Bureau of Statistics
ϕ	Diagnostic rate	0.5–0.75	National Bureau of Statistics and MENET
ϱ	Outpatient rate	0.85	National Bureau of Statistics and MENET
ε	Rate of treatment	0.85	National Bureau of Statistics and MENET
α	Prescription decision coefficient	0.05	Investigation
β	coefficient of patients receiving therapy	0.04	Investigation
ω	Median survival time	5 years	MENET
zz	Retreatment probability of rejected patients	0.05	MENET and investigation
z	Probability of treatment suspension of patients	0.8	MENET and investigation
p	Price of the original drugs	$100,000/two months	MENET and investigation
ψ	Tax rates of original drug/generic drug enterprises	15%	Investigation
τ	Life cycle of patent technologies	10 years	MENET and investigation
d	Substitutability of patent technologies	5%	Investigation

### Research methods

**Fig. 1:**
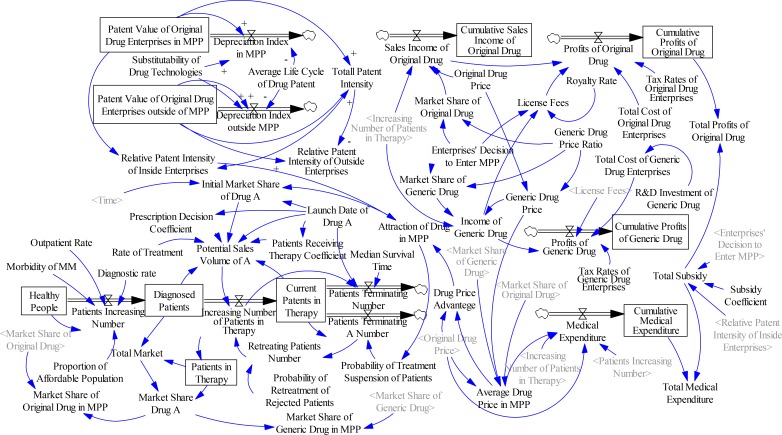
Stock-flow model of enterprises and governments joining the MPP

Key attention was paid to the economic factors influencing the decision of enterprises and governments to join the MPP by balancing economic benefits. To enable calculation of enterprise profits and government medical expenditures, the dynamics model was constructed from three subsystems: drug attraction, dynamic structure of the drug market, and economic performance of the participants. This work assumes that drugs are not monopolized by one original drug enterprise. The MPP abides by the following rules: government balances the initial R&D investment and risks of original drug enterprises by providing subsidies for joining the MPP. After an original drug enterprise joins the MPP, the original drug price may be retained. The drug price of generic drug enterprises should be controlled within a certain range. All technological improvements to the drug patent must be fed back to the original drug enterprises for free. The total stock-flow of enterprises’ and governments’ joining the MPP is shown in Fig. 1.

The validity of the model structure and behaviors was tested, and the model structure and behaviors were proven to be consistent with those of the actual system. In general, the constructed model was reliable. The main subsystem model structures and formulas are described as follows.

### Relative intensity and pooling subsidy coefficient of drug patents

Based on previous research findings, the enterprise innovative index was determined by the R&D investment (18). In this study, the drug patent intensity of different original drug enterprises was hypothesized to be determined by the total R&D investment, and the initial drug patent value (*V_0_*) was determined to be equal to the initial R&D investment. The value of drug patent technology is consumed over time, supposing the existing patent intensity of enterprises decreases at a rate that is proportional to its current level. In other words, patents depreciate exponentially. This depreciation index is related to the life cycle of the patent technology and the substitutability of drug technologies.

[1] $$\frac{dV(t)}{dt}=-\frac{V(t)}{\tau +1/d}$$

where the parameter τ is the average service life of drug patent in relative to technologies and d is the substitutability of drug technologies. The relative patent strength of enterprises is proportional to the cumulative R&D level. The relative patent intensity of original drug enterprises in MPP is *r*_池内_ (*t*) = *V*_池内_ (*t*) / [*V*_池内_ (*t*) + *V*_池外_ (*t*)]. Drug attraction is hypothesized to be determined by the relative drug patent intensity and average price level. According to previous investigations, the amount of pooling subsidy is the product of a pooling subsidy coefficient and the initial R&D investment of the original drug enterprise in the current market. In this study, the initial R&D investment of Celgene Corporation for lenalidomide in the Chinese market was 1/20 of the announced global R&D investment.

### Product market dynamics

**Fig. 2:**
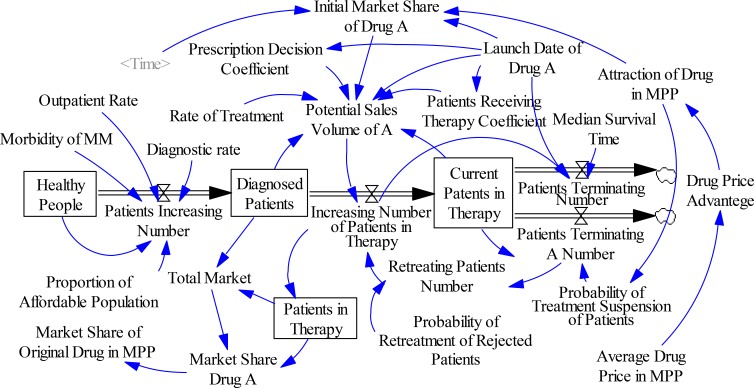
Drug market dynamics model before and after joining the MPP

The drug market dynamics model proposed by Paich et al. was improved to predict the sales volumes of patent drugs (26). The drug market dynamics model before and after enterprises and governments join the MPP is shown in Fig. 2.

In Fig. 2, diagnosed patients (*UP(t)*) become the current patients in therapy (*CP(t)*). The probability of new patients accepting the patent drugs of enterprises in the MPP, the probability of patients undergoing other treatments to change their treatment plan, and the probability of retreatment of rejected patients could be determined by the attraction of the patent drug in the MPP. The dynamic relationship between *UP(t)* and *CP(t)* is:

[2] $$\frac{dUP(t)}{dt}=up(t)-cp(t)$$

[3] $$\frac{dCP(t)}{dt}=cp(t)-ccp(t)-dp(t)$$

where *up(t)*, *cp(t)*, *ccp(t)*, and *dp(t)* are total number of increased patients, number of increased patients accepting the drug treatment, number of patients terminating the treatment, and number of patients terminating all treatments, respectively. Here, *up(t)* is the sum of the potential sales volumes of drugs in unit time and number of retreatment patients. The effects of prescription guidance and patient communication on the potential sales volumes of the launched drugs are expressed by the prescription decision coefficient (*α*) and patients receiving therapy coefficient (*β*). The potential sales volume of a medicine (*pp(t)*) can be calculated as follows:

[4] pp(t)=mδ(t)[αUP(t)+βCP(t)]

In this study, δ(t) is used to express whether the original drug enterprises enter the MPP within a certain period of time; its value is 1 or 0. The parameter m represents the market share of the original drug and its generic drug in patients undergoing treatment. According to investigations, *m* could be hypothesized to be 25% and the market share of generic drugs 6 month after its launch is 0.7*m*.

### Companies’ economic performance and accessibility to medicines

The profits of original and generic drug enterprises that refused to join the MPP before patent expiration are influenced by sales income, drug cost, and marketing cost. The incomes of original drug enterprises joining the MPP before patent expiration were determined by the sales income of a product and the license fees of drug patents. Therefore, the profits of original and generic drug enterprises in the MPP can be calculated. When governments agree to join the MPP, the total medical expenditure is equal to the total volume of the medicinal purchase and subsidies to original drug enterprises. If the government does not agree to join the MPP, the total medical expenditure becomes equal to the total cost of the medicinal purchase.

## Results

### Effect of generic drug price ratio and pooling subsidy coefficient on the decision of enterprises and governments to join the MPP

**Fig. 3:**
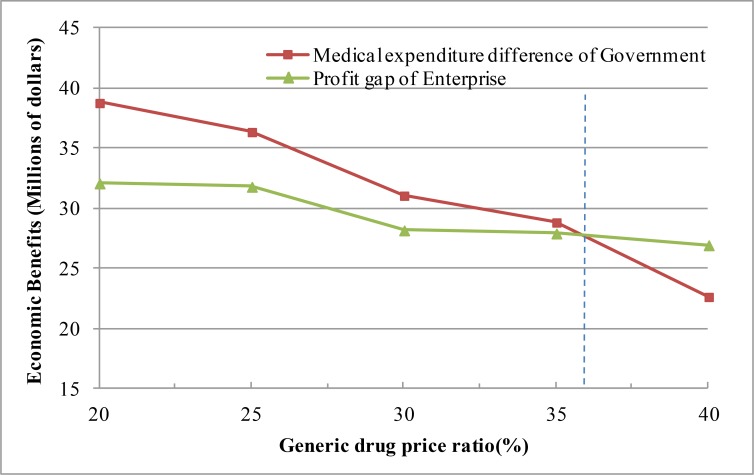
Effects of generic drug price ratio on the economic benefits of original drug enterprises and governments

Profits changes of stakeholders were investigated and compared under different generic drug price ratios and pooling subsidy coefficients to select the appropriate subsidy strategy. Profits and medical expenditure differences under different generic drug price ratios were calculated, and the results are shown in Fig. 3. These results can be described as follows:

1) When the generic drug price ratio is less than 35.8, the government subsidy for original drug enterprises brings about higher benefits. By contrast, the total government medicine expenditure remains lower than the total fiscal expenditure when original drug enterprises do not join the MPP.
2) When the generic drug price ratio is equal to 35.8, the disbursement balance of the government medical source is equal to the profit gap of enterprises.
3) When the generic drug price ratio is greater than 35.8, the government subsidy for original drug enterprises is consistently lower than the profit difference of enterprises; by contrast, the total government medical expenditures are consistently higher than that before original drug enterprises join the MPP. This finding reveals that governments cannot balance the profit loss of original drug enterprises by providing a subsidy lower than the medical expenditure difference.

**Fig. 4:**
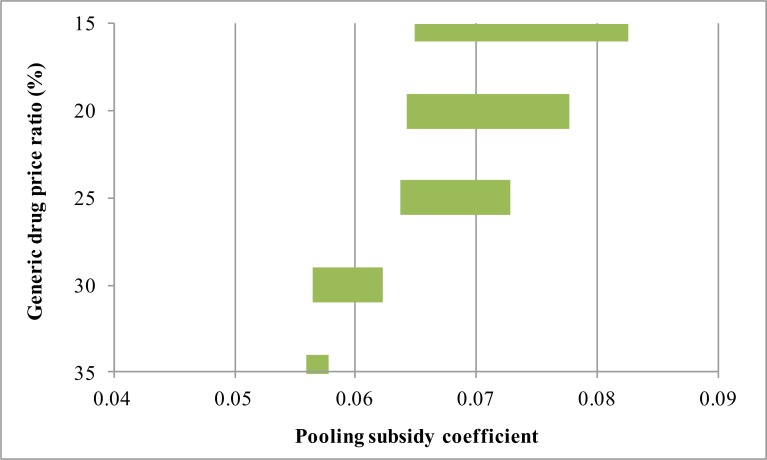
Effects of generic drug price ratio on the economic benefits of original drug enterprises and governments

The subsidy coefficient interval when the generic drug price ratio ranges between 15%–35% was analyzed, and the results are shown in Fig. 4. As the generic drug price ratio is reduced, the interval for the pooling subsidy coefficient gradually increases and the scope of agreement slowly expands. In the actual situation, these parameters are correlated with the market structure and prices of different drugs.

### Effects of royalty rate in the MPP on generic drug enterprises and governments

**Fig. 5:**
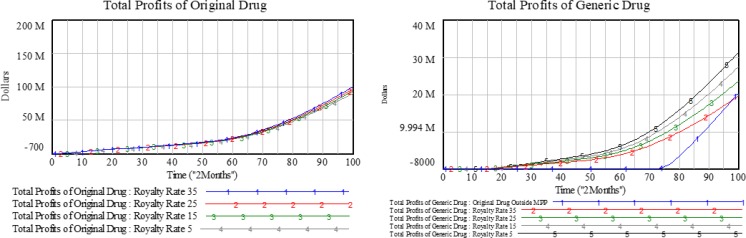
Effects of different royalty rates on different enterprises

After original drug enterprises join the MPP, generic drug enterprises pay a certain fee for patent use. Supposing the drugs prices remain constant, the price of generic drugs cannot be increased beyond a certain range. In this study, the effects of license fees in the MPP were calculated by setting different royalty rates; the results of this analysis are shown in Fig. 5.

Since license fees only account for part of the profits of original drug enterprises, they may influence the total profits only slightly and present no impacts on government medical expenditures. However, license fees influence the profits of generic drug enterprises significantly. When the royalty rate is higher than 34%, the cumulative profits of generic drug enterprises in the MMP decrease.

### Effects of R&D intensity and scale of original drug enterprises on their decision to join the MPP

**Fig. 6:**
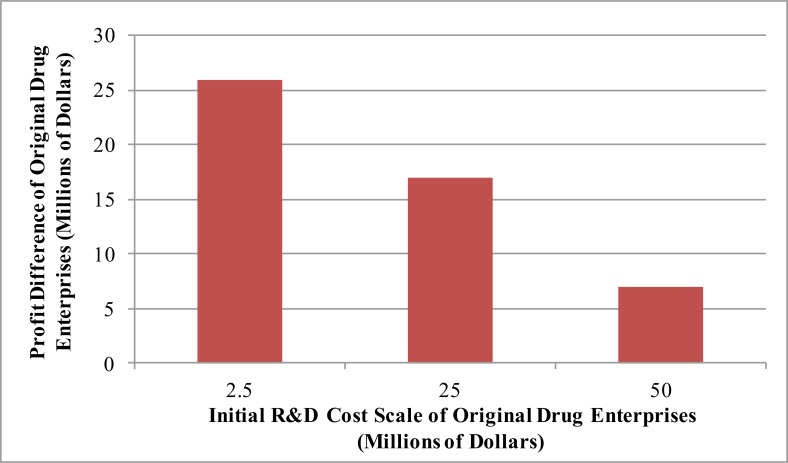
Effects of the initial R&D cost scale of original drug enterprises on the profit difference

**Fig. 7:**
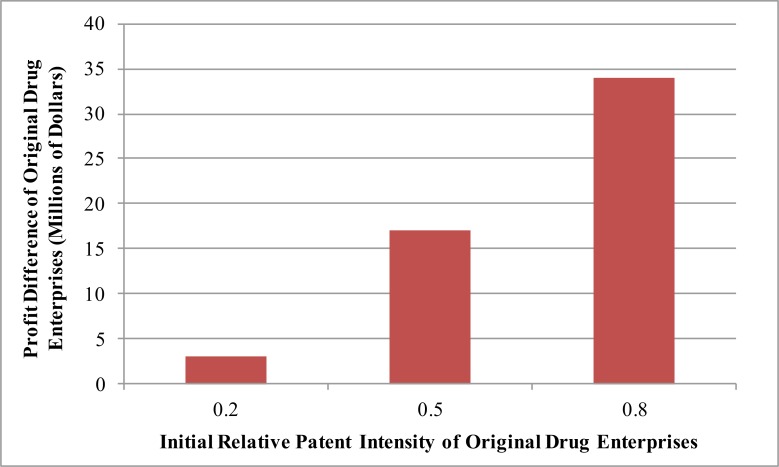
Effects of the initial relative patent intensity of original drug enterprises on the profit difference

The early R&D investment scale and relative drug patent intensity of original drug enterprises may influence their decisions to join the MPP. The influences on enterprise profits are shown in Figs. 6 and 7, respectively.

The total initial cost of original drug enterprises is hypothesized to change, while the ratio between the R&D cost of the original drug enterprise in MPP and the competitors (i.e., other original drug enterprises) is constant. As R&D costs increase, the profit difference before and after joining the MPP gradually declines if the market capacity is constant. The initial investment of original drug enterprises exerts negative impacts on the decision to join the MPP.

Suppose the initial R&D cost of original drug enterprises who decided to join the MPP is constant. Lower the relative patent intensity ratio weakens the drug attraction, thereby results in a smaller market share within the MPP than outside of it. A lower profit difference translates into a higher likelihood of a original drug enterprises joining the MPP.

## Discussions

The research results demonstrate that low-enough generic drug prices are a prerequisite for governments joining the MPP. When the generic drug price is low, medical expenditures decrease after a government joins in. An appropriate pooling subsidy coefficient and royalty rate may drive stakeholders’ decision to join the MPP. Certain pooling subsidy coefficients can help achieve balance between original drug enterprises and governments in the MPP. An appropriate royalty rate may offset the economic loss suffered by original drug enterprises in the MPP while protecting the profits of generic drug enterprises (6).

With constant market incomes, original drug enterprises with a larger initial scale and a lower relative intensity in the whole market are more willing to join the MPP than other types of enterprises. This finding indicates that large-scale non-monopoly innovative enterprises are likely to join the MPP. Joining the MPP could balance the expenditures and benefits of the organizations with high-investment and expand the market shares of the businesses with low patent intensity (19).

Medical innovations brought by MPP can produce high-quality medical resources at a lower cost and protect the public health system. This study provides some suggestions on this area. First, medical management departments should adjust generic drug prices to a reasonable low-price interval and improve public knowledge on generic drugs through technological support and financial encouragement. Second, medical management departments should be more transparent about drug R&D costs while increasing the subsidy scale to encourage more original drug enterprises to join the MPP (29). It is suggested to combine with the definitions of enterprise obligations and compulsory license negotiation strategies to encourage the accession of original drug enterprises, as well as increase medical quality and efficiency of medical organizations (30). Third, after strict approval of the qualifications of an enterprise, the MPP fund may offer preferential policies to larger international original drug enterprises or non-monopoly innovative medical enterprises. In addition, the license fees of drugs can be increased to some extent under the premise of adequate profits to generic drug enterprises, aiming to balance part of the government subsidy or transfer into government fiscal revenues through taxation.

## Conclusion

Increasing the involvement of governments and enterprises in the MPP facilitates the sharing and innovation of medical resources. In this study, a system dynamics model of the drug market was constructed. The effects of the key factors influencing the stakeholders’ decision were analyzed based on the MENET database and annual report of Celgene Corporation. The study shows that appropriate pooling subsidy coefficients and royalty rates may encourage enterprises and governments join the MPP, when the generic drug price is low enough. Moreover, original drug enterprises with higher R&D costs and lower relative patent intensities are likely to join the MPP. The research findings provide medical management departments a reference for formulating medical policies. However, this study presents a limited sample data region. Future studies can classify essential drugs in the MPP.

## Ethical considerations

Ethical issues (Including plagiarism, Informed Consent, misconduct, data fabrication and/or falsification, double publication and/or submission, redundancy, etc.) have been completely observed by the authors.
